# Migration Dynamics of Human NK Cell Preparations in Microchannels and Their Invasion Into Patient‐Derived Tissue

**DOI:** 10.1111/jcmm.70481

**Published:** 2025-03-30

**Authors:** Alina Moter, Sonja Scharf, Hendrik Schäfer, Tobias Bexte, Philipp Wendel, Emmanuel Donnadieu, Martin‐Leo Hansmann, Sylvia Hartmann, Evelyn Ullrich

**Affiliations:** ^1^ Goethe University Frankfurt, Department of Pediatrics, Experimental Immunology and Cell Therapy Frankfurt (Main) Germany; ^2^ Goethe University Frankfurt, Frankfurt Cancer Institute (FCI) Frankfurt (Main) Germany; ^3^ Institute of Pathology University Hospital Essen, University of Duisburg‐Essen Essen Germany; ^4^ Institute for Transfusion Medicine and Immunohematology German Red Cross Blood Service Baden‐Württemberg – Hessen Hessen Germany; ^5^ Goethe University Frankfurt, University Cancer Center Frankfurt (UCT), University Hospital Frankfurt Frankfurt (Main) Germany; ^6^ Institute for Organic Chemistry and Biochemistry Technical University of Darmstadt Darmstadt Germany; ^7^ German Cancer Consortium (DKTK) Partner Site Frankfurt/Mainz and German Cancer Research Center (DKFZ) Heidelberg Germany; ^8^ Universite' Paris Cité, CNRS, INSERM Equipe Labellisée Ligue Contre le Cancer, Institut Cochin Paris France; ^9^ Frankfurt Institute for Advanced Studies (FIAS) Frankfurt (Main) Germany; ^10^ Institute of General Pharmacology and Toxicology Goethe University Frankfurt (Main) Germany

**Keywords:** CAR‐NK, DLBCL tissue, hyperplastic lymphoid tissue, microchannel, motility, NK cells, T cells

## Abstract

Natural killer (NK) cells are characterised by their ability to attack cancer cells without prior antigen stimulation. Additionally, clinical trials revealed great potential of NK cells expressing chimeric antigen receptors (CARs). Successful anti‐tumour efficacy remains limited by migration and infiltration to the tumour site by NK cell preparations, which is linked to the scarcity in the knowledge of migration dynamics and invasion potential. Here, we applied a recently reported innovative microfluidic microchannel technology to gain insight into the intrinsic motility of NK cells. We assessed the baseline activated and proliferating NK cells in direct comparison with T cells and investigated their motility patterns in the presence of tumour cells. Additionally, we performed high‐resolution 4D confocal imaging in patient‐derived hyperplastic lymphatic tissues to assess their invasive capacity. Our data revealed that the invasion potential of NK cells was greater than that of T cells, despite their similar velocities. The flexibility of the NK cell nucleus may have contributed to the higher invasion potential. The motility of CD19‐CAR‐NK cell preparations was similar to that of non‐transduced NK cells in hyperplastic lymphoid tissue, with improved targeted migration in tumour tissue, suggesting the suitability of genetically engineered NK cells for difficult‐to‐reach tumour tissues.

## 1 Introduction

Natural killer (NK) cells act as the first line of defence, recognising and eliminating virus‐infected and malignant cells [1]. Unlike T cells, which require prior T‐cell receptor activation, NK cells can recognise tumour cells without prior antigen stimulation by regulation through a broad repertoire encoding for activating (e.g. NKG2D, DNAM‐1 and NCRs) or inhibitory (e.g. KIR receptors and NKG2A/B) receptors [1, 2]. Furthermore, the killing capacity of NK cells is partially regulated by CD16, which mediates antibody‐dependent cellular cytotoxicity (ADCC) [1, 2].

For T cells, in addition to their mode of action, migration behaviour in the absence or presence of tumour cells has been well studied in recent years [3, 4, 5, 6, 7, 8], whereas for NK cells, only limited data on motility and tissue infiltration capacity are available [9, 10, 11]. Most motility studies have been performed using murine NK cells, which also allow intravital two‐photon microscopy to be performed in situ in healthy mouse lymphoid tissue, stromal cells, or lymphoma‐bearing mice [12, 13]. To date, only one study by Bajénoff et al. [14] has reported a comparative motility analysis of murine T and NK cells in mice and observed that NK cells exhibit slower motility than T cells [14].

Little is known about the migratory and invasive potential of human NK cells. The first reports on human NK cell motility were based on microchip imaging assays or confocal microscopy, and heterogeneous velocity and motility patterns were observed in resting NK cells, cytokine‐activated NK cells, or in the presence of stromal cells [9, 10].

Therefore, in this study, we aimed to apply an innovative technology using microfluidic microchannel techniques to study NK cell motility and velocity in comparison with those of T cells. Microfluidic microchannel assays can be used to assess cell motility and velocity in vitro. The velocity of murine T cells in microchannels with varying T cell densities was studied to evaluate the impact of microchannel width and media tonicity on their motility [8]. Moreover, Xu and Pang [11] demonstrated that human NK‐92MI cells exhibited faster velocity in straight microchannels than on flat surfaces when co‐cultured with breast cancer cells [11]. Here, we established an experimental microchannel setting to directly compare the motility and velocity of human primary expanded NK and T cells in the presence of B‐cell acute lymphoblastic leukaemia (B‐ALL) and diffuse large B‐cell lymphoma (DLBCL) cells based on a microchannel system recently reported by our group [5, 15]. In addition, ex vivo 4D imaging of fresh slices made from patient‐derived hyperplastic lymphatic tissues was used to closely mimic the movement pattern of NK and T cells in an intact tissue microenvironment and to investigate their invasion and penetration behaviour.

Clinical trials have reported promising outcomes for the treatment of patients with B cell leukaemia and lymphoma with CD19‐chimeric antigen receptor (CAR) NK cells [16, 17]. We recently developed non‐viral technologies for the genetic engineering of NK cells, such as Sleeping Beauty (SB) transposon‐based technology, which led to CAR‐NK cell preparations with high anti‐leukaemic efficacy [18, 19, 20, 21, 22].

The aim of the present study was to further investigate the motility and tissue invasion behaviour of non‐virally engineered CD19‐CAR‐NK cells compared to non‐transduced (NT) NK cells in microchannels as well as within patient‐derived hyperplastic lymphatic and DLBCL tumour tissues.

## 2 Materials and Methods

### 2.1 Isolation and Cultivation of Immune Cells

NK and T cells were isolated from the buffy coats of healthy donors provided by the German Red Cross Blood Donation (DRK‐Blutspendedienst Baden‐Württemberg‐Hessen, Frankfurt am Main, Germany). Peripheral blood mononuclear cells (PBMCs) isolation from whole blood was done by Ficoll density gradient. This study was approved by the Ethics Committee of Goethe University, Frankfurt, Germany (approval no. 329/10).

NK cell isolation and subsequent purity assessment were performed as previously described [18, 20, 21]. T cells were cultivated according to previously reported protocols by Pommersberger et al. [23] Additional details regarding the isolation and cultivation of NK and T cells are provided in Supplementary Information.

After 7–21 days of expansion, motility analysis was performed using a microchannel assay within the hyperplastic lymphoid tissue. One day prior to the analysis, NK and T cells received half of the medium change. Prior to motility analysis, the purity and phenotype of NK and T cells were assessed using flow cytometry, as described in the Supplementary Information.

### 2.2 Generation of SB‐Based CD19‐CAR‐NK Cells

CD19‐CAR‐NK cells were generated from primary NK cells using a non‐viral SB transposon/transposase‐based system, as previously reported by our group [20]. Briefly, 1 × 10^6^ primary NK cells were nucleofected by using 2.5 μg SB100X mRNA (kindly provided by Ethris GmbH, Planegg, Germany) and 1 μg CD19‐CAR minicircle DNA (PlasmidFactory GmbH&Co. KG, Bielefeld, Germany). Nucleofection was performed using the P3 Primary Cell 4D‐Nucleofector Kit S and 4D Nucleofector (both Lonza, Basel, Switzerland). After nucleofection, the cells were transferred to the cultivation medium and cultivated as described above. The cells were cultivated for an additional 18–25 h before performing the functional assays.

### 2.3 Cytotoxicity Analysis of Non‐modified NK Cells

Cytotoxicity assays were performed as described previously [18, 19, 20, 21]. Briefly, to measure the cytotoxic functionality of non‐modified NK cells, CellTrace CFSE‐stained tumour cells (NALM‐6 and TMD8) were co‐cultivated with NK cells at effector‐to‐target (E:T) ratios of 1:1 and 2:1 and incubated at 37°C with 5% CO_2_ for 20 h. The cytotoxic functionality of CD19‐CAR‐NK cells in comparison to that of NT NK cells was assessed, as described in the Supplementary Information.

### 2.4 Microchannel Assay

Polydimethylsiloxane (PDMS) chips with different types of microchannels were produced in moulds provided by Dr. Matthieu Piel, Institut Curie [24]. Straight channels with a diameter of 4 μm and a height of 10 μm were tested and determined to be the most appropriate in size. Non‐modified NK and T cells (after 14–21 days of ex vivo expansion) or CAR‐NK and NT NK cells were stained with CellTrace Violet Proliferation Kits (Thermo Fisher Scientific, Waltham, MA, USA), and then 2 × 10^5^ cells were seeded in a microchannel punch. The other punch on the opposite side of the microchannels was loaded with 2 × 10^7^ NALM‐6 or TMD8 cells, which were previously stained with MitoTracker Deep Red according to the manufacturer's protocol (Cell Signalling Technology, Danvers, MA, USA). Cell motility was monitored using a fluorescence microscope (Lumascope LS720; Etaluma, Carlsbad, CA, USA) in an incubator for 20 h, and time‐lapse images were taken every 4 min in straight channels. The cells in the videos from the microchannel experiments were segmented using a custom script as previously described [15].

### 2.5 Ex Vivo 4D‐Imaging of Tissue

For 4D‐imaging, native thick sections of hyperplastic lymphatic tissue from the pharyngeal tonsils and DLBCL tissues were analysed as previously described [25, 26, 27]. The study was conducted in accordance with the Declaration of Helsinki after obtaining written informed consent from all patients and was approved by the ethics committee of Goethe University Hospital (Nr20‐876aV[Frankfurt] and 24‐12237‐BO [Essen]). In brief, fresh hyperplastic lymphoid tissues (tonsils) or DLBCL tissue were embedded in low‐melting‐point agarose, and 400 μm slices were generated with a vibratome, and stained and imaged as described in the Supplementary Information.

### 2.6 Nuclear Lamin Staining of NK and T Cells

Cytospins of NK and T cells were produced according to standard conditions and incubated with a monoclonal anti‐lamin A/C antibody (1:200; sc‐7292; Santa Cruz Biotechnologies, Dallas, TX, USA), as previously described [15]. For detection, the VectaFluor Excel Antibody Kit Dylight 594 (Vector Laboratories, Burlingame, CA, USA) was used, and 3D imaging was performed with a Leica TCS SP8 confocal microscope (Leica Microsystems, Wetzlar, Germany). The settings used for confocal microscopy are described in the Supplementary Information.

### 2.7 Statistics

Statistical analyses were performed using GraphPad Prism version 10.2.3 (La Jolla, CA, USA). The number of cells entering the microchannel or penetrating the adenoid tissue was statistically assessed using mixed‐effects analysis with Tukey's multiple comparison tests, as values were missing for completely random reasons. To evaluate the velocity statistics from the microchannel assay, as well as the velocity, track length, and displacement from 4D ex vivo imaging of the adenoid tissue, the weighted average of the respective values for each donor was calculated. Data were statistically analysed using the Wilcoxon matched‐pairs signed‐rank test (paired and non‐parametric) with a confidence level of 95%. Significance was set at *p ≤ 0.05, **p ≤ 0.005, and not significant (ns) *p* > 0.05 (*p* values are indicated).

## 3 Results

### 3.1 NK Cells Differ From T Cells in Their Capability to Enter Microfluidic Microchannels

**FIGURE 1:**
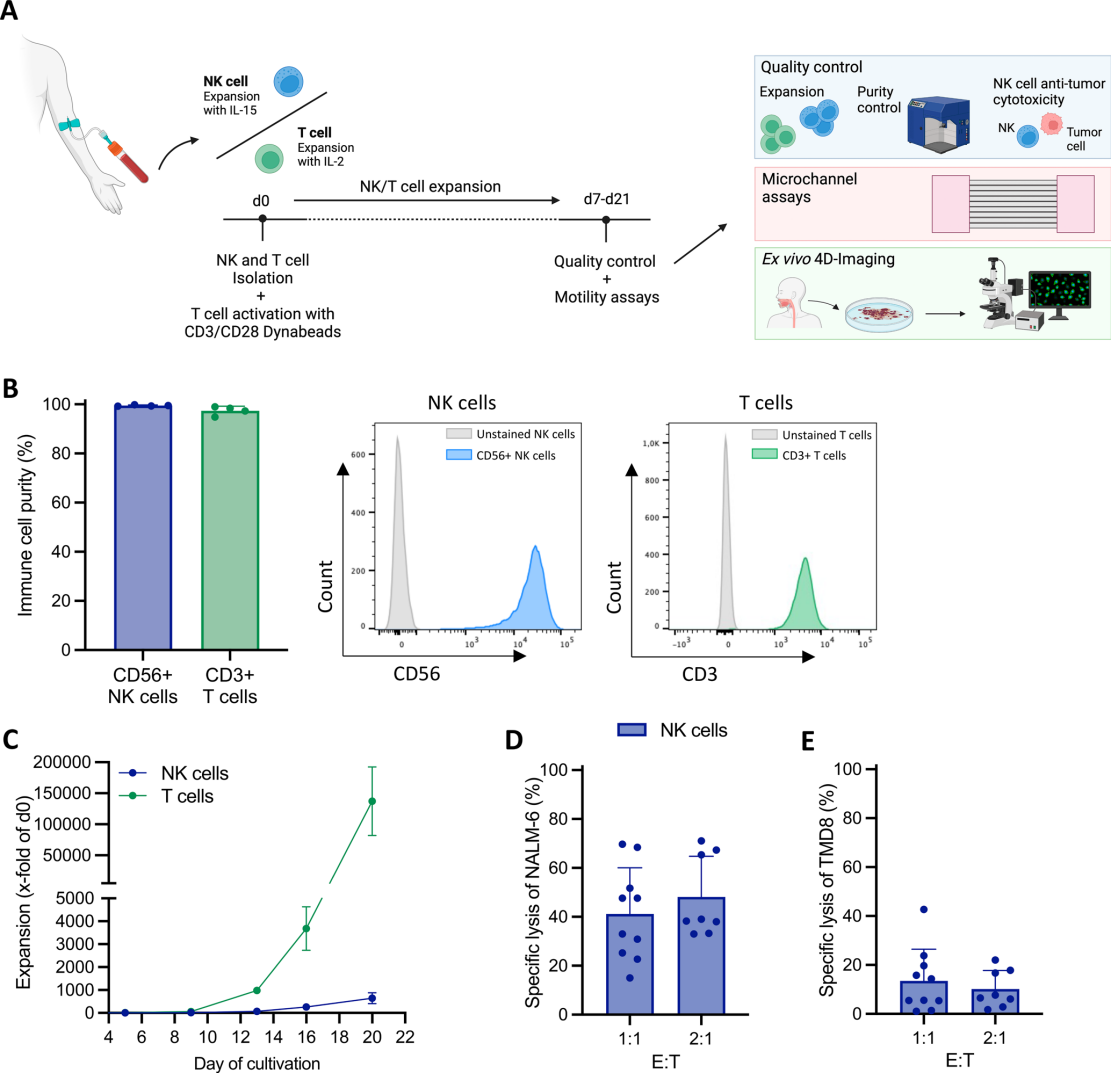
Expansion and functionality of NK and T cells. (A) Graphical representation of the study workflow: NK and T cells were isolated from identical healthy human donors (buffy coats) and expanded for 7–21 days. Following quality controls, analysis of cell motility using a microchannel‐based assay and ex vivo 4D‐imaging of patient‐derived adenoid tissue was performed. (B) Flow cytometry‐based evaluation of cell purity of CD56^+^ NK and CD3^+^ T cells (*n* = 4). (C) Expansion of NK and T cells (*n* = 2). (D, E) Cytotoxic anti‐tumour functionality of NK cells against (D) NALM‐6 and (E) TMD8 at an E:T ratio of 1:1 and 2:1 after 20 h of co‐cultivation (*n* = 8–10).

In the first approach, the motility and velocity of human NK cells were compared to those of T cell preparations using microfluidic microchannels based on protocols recently reported for lymphoma and T cells [5, 15]. Prior to the motility assessment, phenotypic and functional quality control of the immune cell preparations was performed. Briefly, human NK and T cells were isolated from the same healthy donors and cultured under optimised conditions with respect to the immune cell type. NK cells were cultured with IL‐15 every 3–4 days. In contrast, T cells were stimulated with CD3/CD28 dynabeads for 6–7 days and cultivated with 50 IU/mL IL‐2 every 3–4 days. NK and T cells were cultured for 7–21 days, depending on the assay performed, and were stimulated with half of their respective cytokines on the day before the motility analyses (Figure 1A).

For quality control, the purity and phenotype of the ex vivo expanded NK and T cell preparations, as well as their proliferative expansion, were carefully assessed (Figure 1B,C). Assessment of the phenotype confirmed pure CD56^+^ NK cells, which consisted of 80.18% CD16^high^ and 19.33% CD16^dim^ NK cells, and pure CD3^+^ T cell populations, with 55.17% CD8^+^ and 40.60% CD4^+^ T cells (Figure 1B and Figure S1A,B). Consistent with our expectations, T cells outperformed NK cells in terms of expansion potential (Figure 1C).

Because NK cells are expected to mediate anti‐tumour cytotoxicity without antigen stimulation, in vitro cytotoxicity analyses were performed to evaluate the intrinsic killing capacity of IL‐15 expanded NK cell preparations in parallel with the planned motility analyses. This assay was conducted for the same duration as the motility analyses, with both experiments lasting for 20 h (Figure 1D,E).

**FIGURE 2:**
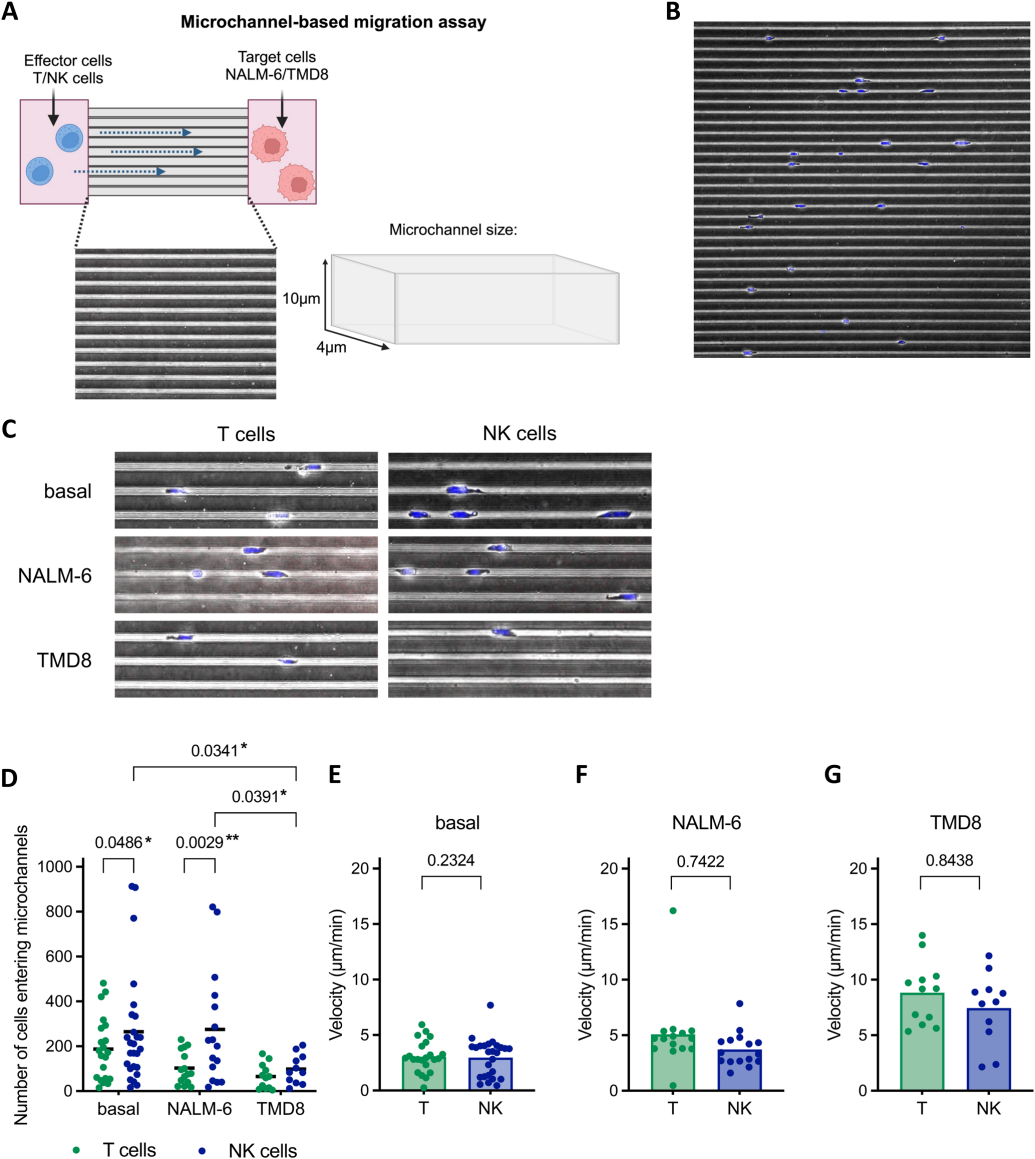
NK and T cell motility in the presence of NALM‐6 and TMD8 in fibronectin‐coated microchannels. (A) Schematic illustration of the microfluidic microchannel assay. NK/T cells and tumour cells (NALM‐6 or TMD8) were seeded in their respective reservoir of the microchannels. NK/T cell motility was measured for 20 h with 4 min imaging intervals. Exemplary images of (B) the microchannel frame and (C) NK and T cells in the microchannel under basal conditions or in the presence of tumour cells. (D) The number of NK and T cells entering the microchannel under basal conditions (*n* = 10) or in the presence of NALM‐6 (*n* = 8) or TMD8 (*n* = 6). One donor is represented by *n* = 1–3 data points. Velocity (μm/min) of NK and T cells under (E) basal condition (*n* = 10), in presence of (F) NALM‐6 (*n* = 8) or (G) TMD8 (*n* = 6). One donor is represented by *n* = 1–3 data points. Statistical analysis was performed using a mixed‐effects model with Turkey's multiple comparison (number of tracks) or Wilcoxon matched‐pairs signed rank test (velocity). Statistics for the velocity data were calculated using the weighted averages of all donors. Statistical significance thresholds were set to *p ≤ 0.05, **p ≤ 0.005, ns *p* > 0.05 (*p* values are indicated), (D) if not indicated no significant differences were observed.

Microchannels were prepared on PDMS chips and coated with fibronectin. To determine the optimal microchannel width for NK and T cells, we optimised the microchannel size based on previous studies [5, 15]. Straight channels with a height of 10 μm and a width of 4 μm were found to be the most appropriate size, as one cell exactly fits within the diameter of the channel (Figure 2A). NK and T cell motility was assessed for 20 h at an imaging interval of 4 min under basal conditions (absence of stimuli) and in the presence of the B‐ALL cell line NALM‐6 or the DLBCL cell line TMD8. We analysed the rate of entry into the microchannel and the velocities of the NK and T cells (Figure 2A). Exemplary images show the microchannel frame (Figure 2B and Movie S1) as well as NK and T cells in the microchannel under basal conditions and in the presence of NALM‐6 and TMD8 (Figure 2C).

The mean number of cells entering the microchannels was significantly higher for NK cells, with a mean of 264.65 cells entering, compared to a mean of 187.95 T cells under basal conditions (*p* = 0.0486). In the presence of NALM‐6, the entry rate of NK cells remained higher (mean 275.50 cells) than that of T cells (mean 102.87, *p* = 0.0029) (Figure 2D). However, in the presence of TMD8 in the microchannels, both NK and T cells showed reduced entry rates (means 98.27 cells and 65.08, respectively). The difference in the number of NK and T cells entering the microchannels in the presence of TMD8 was not significant (Figure 2D).

Under basal conditions, NK cells demonstrated a mean velocity of approximately 3.0 μm/min, which was comparable to that of T cells (Figure 2E). In microchannels containing NALM‐6, NK cells moved with a mean velocity of 3.72 μm/min, while T cells tended to move faster (mean 5.09 μm/min) (Figure 2F). The presence of TMD8 led to an increase in motility for both cell types, with NK cells reaching a mean velocity of 7.44 μm/min and T cells achieving an acceleration to a mean of 8.82 μm/min (Figure 2G).

These findings indicate that NK cells achieve higher frequencies of entry into microchannels, whereas NK cells and T cells move at similar velocities.

### 3.2 Ex Vivo 4D‐Imaging of Patient‐Derived Hyperplastic Lymphatic Tonsil Tissue Shows Higher Invasion and Penetration of NK Cells

**FIGURE 3:**
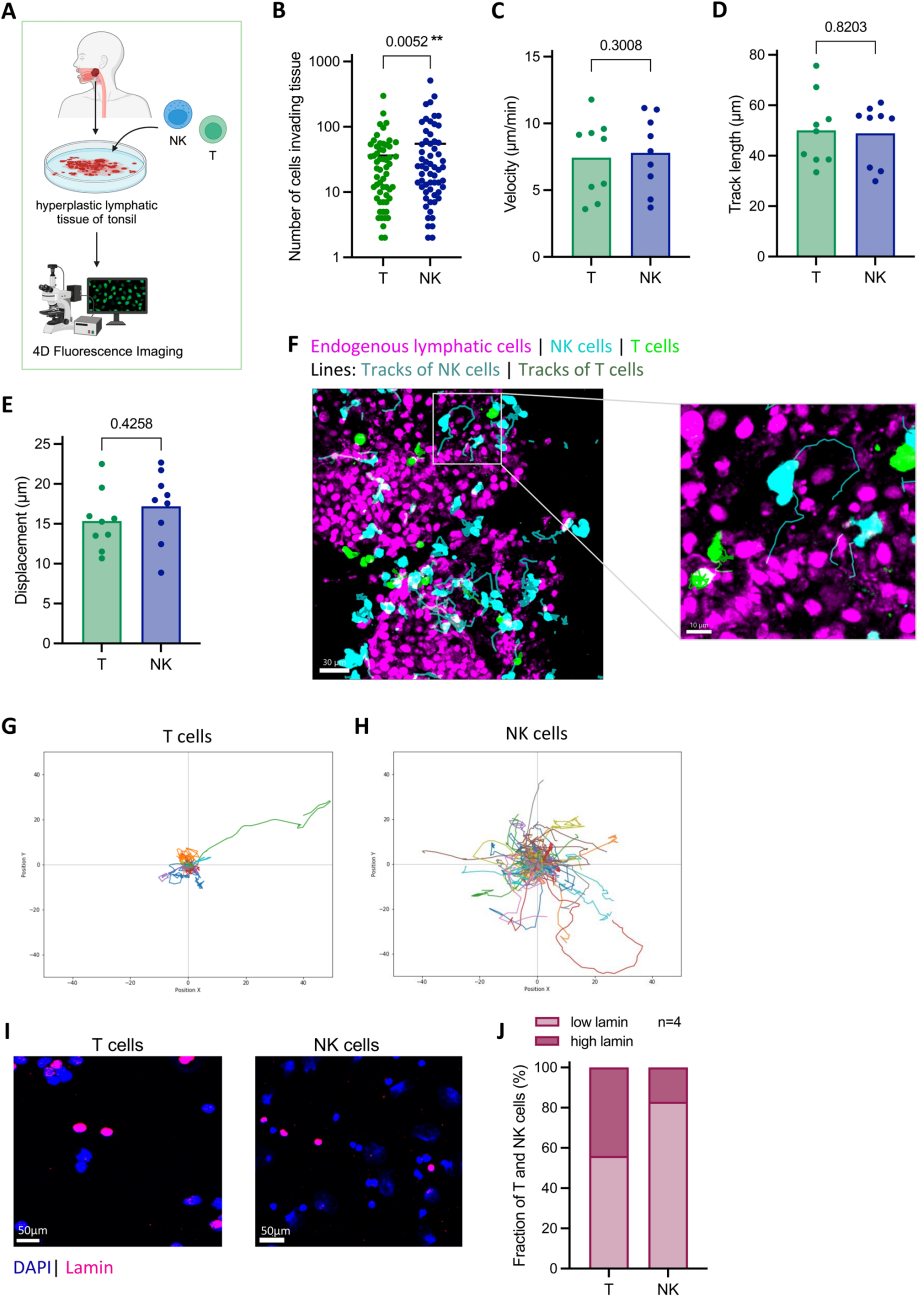
NK and T cell motility in patient‐derived human hyperplastic lymphatic tonsil tissue. (A) Schematic illustration of ex vivo 4D‐imaging of patient‐derived human hyperplastic lymphatic tonsil tissue. NK and T cells were added simultaneously to the tissue slices and imaged using 4D fluorescence microscopy. Videos were taken from different sections of the same tissue slice (*n* = 4–8), and all visible NK and T cells were tracked. (B) Number of NK and T cells infiltrating the tissue. Each data point represents data from a single video (*n* = 4–8), taken of a distinct section of a certain tissue slice with NK and T cells (*n* = 9). (C) Velocity (μm/min) of NK and T cells in hyperplastic lymphatic tonsil tissue from multiple videos (*n* = 4–8) of different sections of the same tissue slice. (D) Track length (μm) and (E) displacement (μm) of NK and T cells in hyperplastic lymphatic tonsil tissue. (C–E) Each data point represents the weighted average of all tracked cells, derived from one NK or T cell donor (*n* = 9) from multiple videos (*n* = 4–8) of different sections of the same tissue slice. (F) Exemplary image of adenoid tissue slice with endogenous CD19^+^ lymphatic cells (magenta) following addition of primary NK (blue) and T cells (green). Tracks of NK and T cells are indicated by thin lines. Individual tracks of (G) T cells and (H) NK cells from one representative donor tracked in hyperplastic lymphatic tonsil tissue. (I) Representative image of NK and T cells stained with DAPI (blue) and an anti‐lamin A/C antibody (magenta). (J) Percentage distribution of NK and T cells (*n* = 4) showing a high or low area of nuclear lamin. The cut‐off for low lamin was set to ≤ 30% and for high lamin to > 30%. Statistical analysis was assessed using the Wilcoxon matched‐pairs signed rank test for the number of tracks, velocity, track length, and displacement. Statistical significance thresholds were set to *p ≤ 0.05; **p ≤ 0.005; ns *p* > 0.05 (*p* values are indicated).

To further validate the results of the microchannel assay, we conducted ex vivo 4D imaging of patient‐derived hyperplastic lymphatic tissue slices to closely mimic the in vivo situation and movement patterns of NK cells within the human body (Figure 3A). Prior to imaging, NK and T cells loaded with fluorescent dyes were incubated on the tissue slices for 1 h. The slices were then rinsed, and fluorescent NK and T cells were tracked in 2–8 sections of each tissue using dynamic confocal microscopy.

The ex vivo imaging results closely matched the microchannel data, showing that an average of 54.97 NK cells penetrated the tissue, which was significantly more than the average of 36.38 T cells observed (*p* = 0.0052) (Figure 3B). Despite this difference in penetration, no significant difference were observed in the velocity between NK and T cells, with both moving at means of 7.79 μm/min and 7.44 μm/min, respectively (Figure 3C).

Additionally, we analysed the motility of NK and T cells in tissues using track length, displacement, and individual tracks. The mean track lengths of NK and T cells were comparable, measuring 48.91 μm and 50.05 μm, respectively (Figure 3D and Movie S2). However, NK cells showed a slight trend towards increased displacement, with an average of 17.19 μm compared to 15.36 μm for T cells (Figure 3E). Individual tracks from a representative single donor highlighted a more dynamic motility pattern for NK cells than for T cells (Figure 3F–H), a trend that was also visually supported by the representative images (Figure 2F).

We hypothesised that the observed differences in tissue penetration may be due to differences in nuclear stiffness, as previously suggested for other cells [28]. To investigate this, we stained nuclear lamin A/C and found a lower percentage of this area in NK cells than in T cells (Figure 3I). The measured percentage area of the nuclear lamin fraction was classified as high (> 30%) or low (≤ 30%). A proportion of T cells (44%) exhibited high lamin levels, whereas only 17% of NK cells expressed high lamin levels (Figure 3J). Overall, most NK cells (83%) showed a low frequency of lamin A/C expression.

In conclusion, our data support the hypothesis that NK cells possess a higher capacity for tissue invasion and penetration than T cells do. The reduced expression of lamin A/C in the nuclei of NK cells suggests enhanced flexibility, which potentially facilitates their penetration through tissue structures.

### 3.3 Motility of CD19‐CAR‐NK Cells in Microfluidic Microchannels and Patient‐Derived Human Hyperplastic Lymphatic Tonsil Tissue

**FIGURE 4:**
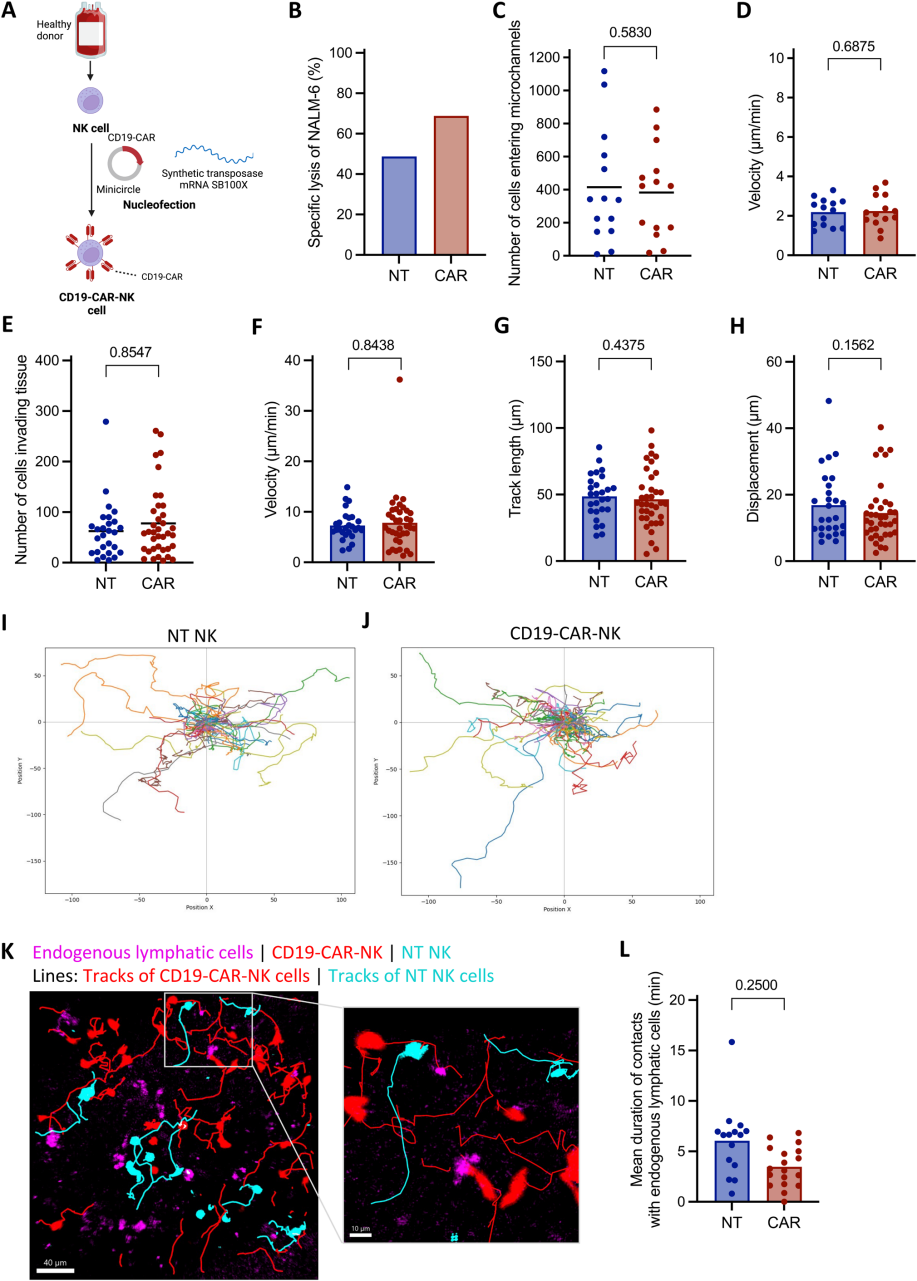
Motility of CD19‐CAR‐NK and NT NK cells under basal conditions in microchannel and in patient‐derived human hyperplastic lymphatic tonsil tissue. (A) Schematic illustration of the workflow for SB‐generated CD19‐CAR‐NK cells. (B) Anti‐tumour functionality of CD19‐CAR‐NK cells from one representative donor after 4 h of co‐culture with NALM‐6. (C) The number of cells entering the microchannel under basal conditions (*n* = 7). One donor is represented by *n* = 2 data points. (D) Velocity (μm/min) of CD19‐CAR‐NK and NT NK cells under basal conditions (*n* = 7) in microchannels. Each donor is represented by *n* = 2 data points. (E) Number of CD19‐CAR‐NK and NT NK cells infiltrating patient‐derived human hyperplastic lymphatic tonsil tissue (*n* = 6). Each data point represents the number of infiltrating cells from a tissue section over the course of one video. (F) Velocity of CD19‐CAR‐NK and NT NK cells in μm/min (*n* = 6). (G) Track lengths of CD19‐CAR‐NK and NT NK cells in μm (*n* = 6). (H) Displacement of CD19‐CAR‐NK and NT NK cells in μm (*n* = 6). (E‐H) Each donor is shown as *n* = 2–8 data points, representing for technical replicates performed on different sections of the same tissue slice. Individual tracks from one representative donor of (I) NT NK cells and (J) CD19‐CAR‐NK cells. (K) Exemplary images of a tissue slice containing endogenous CD20^+^ cells (magenta), CD19‐CAR‐NK cells (red) and NT NK cells (blue). Individual tracks of CAR‐NK and NT NK cells are indicated as thin lines (red and blue, respectively). (L) Mean duration of contacts with endogenous lymphatic cells in minutes (*n* = 3). Each donor is represented by *n* = 4–7 data points. Statistical analysis of the number of tracks was performed using the Wilcoxon matched‐pairs signed rank test with all single data points. The data on velocity, track length, displacement and duration of contacts were statistically assessed using the Wilcoxon matched‐pairs signed rank test with the weighted average of each donor. Statistical significance thresholds were set to *p ≤ 0.05; ns *p* > 0.05 (*p* values are indicated).

After observing no significant differences in the movement patterns of NK and T cells, we investigated whether CAR‐NK cells exhibited any changes in motility due to the genetic modifications. Therefore, we used primary CD19‐CAR‐NK cells (Figure 4A) genetically modified by SB transposition, as previously described [20]. As part of the functional quality control of CD19‐CAR‐NK cells, an exemplary 4 h cytotoxic analysis from one representative donor demonstrated a high level of anti‐tumour functionality compared to non‐transduced NT NK cells (Figure 4B).

As expected, the microchannel assay data revealed no differences between CD19‐CAR‐NK cells and naive NT NK cells in terms of the number of cells entering the microchannel (means of 382.43 and 414.86 cells, respectively) and their velocity (means 2.19 μm/min for NT NK and 2.25 μm/min for CAR‐NK) (Figure 4C,D) under basal conditions.

To further examine the migratory patterns of CD19‐CAR‐NK and NT NK cells in a tissue context, we conducted ex vivo imaging of patient‐derived human hyperplastic lymphatic tonsil tissues. The motility patterns of both were similar, as indicated by comparable velocities, track lengths, displacements, and individual tracks from a representative single donor (Figures 4E–K and Movie S3). However, a slight trend was observed towards an increased number of CAR‐NK cells invading the tissue (mean of 77.68 cells) in comparison to NT NK cells (mean of 62.22 cells, *p* = 0.8547) (Figure 4E). In addition, CAR‐NK cells showed slightly reduced displacement (mean of 14.52 μm) compared to NT NK cells (mean of 16.89 μm, *p* = 0.1562) (Figure 4H).

Regarding the mean duration of contacts with endogenous lymphatic cells, we observed shorter interactions, with a mean duration of 3.47 min for CAR‐NK cells as opposed to 6.05 min for NT NK cells (*p* = 0.2500) (Figure 4L).

In conclusion, the data presented here show no noteworthy differences in motility between CD19‐CAR‐NK and NT NK cells.

### 3.4 Motility of CD19‐CAR‐NK Cells in the Presence of DLBCL Cells

For a more clinically relevant understanding of CD19‐CAR‐NK cell motion, we further analysed their motility in the presence of DLBCL cells in microchannels and ex vivo tumour tissues (Figure S2A). Therefore, we repeated the microchannel‐based motility assay using CD19‐CAR‐NK and NT NK cells in the presence of TMD8 cells. Here, we observed no significant differences between CAR‐NK and NT NK cells but observed a higher heterogeneous distribution for NT NK cells. However, for NT NK cells, a trend towards increased infiltration was observed. A mean number of 178 CAR‐NK cells and 236 NT NK cells entered the microchannel (*p* = 0.0946), with a mean velocity of 3.42 μm/min and 3.80 μm/min, respectively (*p* = 0.4688) (Figures S2B,C).

Furthermore, the movement of CAR‐NK cells was observed in fresh tumour tissue slices from patient‐derived DLBCL biopsy samples. Tumour tissue slices retain their complex original microenvironment and spatial organisation, thereby enabling the analysis of CAR‐NK cell motility in a more clinically relevant setting. The movement of CD19‐CAR‐NK and NT NK cells was investigated by imaging and analysing five different sections from each tissue sample. As observed in the microchannel assay, non‐modified NK cells tended to be superior in invading tissue in comparison to CAR‐NK cells (mean of 155 and 91, respectively), while no difference was observed in velocity (mean of 4.37 μm/min and 3.33 μm/min, respectively) (Figure S2D,E). Additionally, CAR‐NK cells tended to have a slightly reduced track length (mean of 24.32 μm) and displacement (mean of 6.82 μm) in comparison to NT NK cells (mean of 27.53 and 9.65 μm, respectively) (Figure S2F,G). Subsequently, the refinement ratio was calculated using these data, with the calculation being performed by dividing the track length by the displacement (Figure S2H). The data demonstrated an increased refinement ratio for CAR‐NK cells (mean, 4.10) compared to that for NT NK cells (mean, 3.05). Owing to the low number of patient‐derived tumour tissues (*n* = 2), no statistical analysis was performed.

## 4 Discussion

Collectively, these findings provide new insights into the motility and tissue infiltration capacities of primary human NK cells, including genetically engineered NK cell preparations, particularly in hyperplastic lymphatic and lymphoma tissues. Although the capacity of T cells to migrate and infiltrate tissues has been extensively documented, data on human NK cells are rare [3, 4, 5, 6, 7, 8, 9, 10, 11]. To the best of our knowledge, no comparative data is available on the motility of human NK cells in comparison to T cells from identical donor sources. To address this, we directly compared the motility of NK cells with that of T cells using a microfluidic‐based microchannel assay and 4D confocal imaging within ex vivo patient‐derived human hyperplastic lymphatic tonsil tissue.

Our data demonstrated similar migration velocities of NK and T cells from healthy donors. Previously, a comparison between murine NK and T cell movement following infection with *Leishmania major* reported a significantly increased velocity of T cells inside the lymph nodes compared to that of NK cells [14]. However, in this approach, murine NK and T cells were tracked in vivo following protozoan infection, whereas this study analysed human‐derived immune cell motion using in vitro microchannel assays and ex vivo tissue imaging. Given the different immune responses expected in both settings, the divergent trend towards velocity can be explained. In contrast, previous studies reported that NK‐92MI cells in microchannel‐based settings had velocities similar to our findings for human primary NK cells [11]. Comparable data have been reported for primary T cells in the microfluidic microchannel settings used in this study [5]. Furthermore, we confirmed similar velocities of NK and T cells in patient‐derived human hyperplastic lymphatic tissues. These results are consistent with the velocities observed for T cells in native pharyngeal tonsillar hyperplastic tissues and solid tumour tissues reported previously [5, 6].

In the presence of tumour cells, T and NK cells exhibited comparable increases in overall velocity. However, NK cells showed an elevated infiltration capacity compared to T cells. Nevertheless, it remains challenging to translate these in vitro findings to an in vivo context, as this setting lacks a tumour microenvironment (TME) and spatial organisation [29]. Tissue homing and cell motility within the TME are complex owing to the broad immune landscape of B cells, antigen‐presenting cells, dendritic cells, monocytes, macrophages, and neutrophils. Immune cell subsets can have immunosuppressive or supportive effects on cell motility, tissue homing, and the immune response [30, 31, 32]. When comparing the velocities of various immune cell types in the TME from studies reported by others, our findings confirmed similar velocities of T and NK cells, as observed for B cells in in vitro migration assays [33]. In contrast, the macrophages move at a slower rate [34].

In addition, the migration of immune cells in the TME is dependent on proteoglycans and integrins, such as ICAM‐1, VCAM‐1, LFA‐1, and MCA‐1, which can bind to the extracellular matrix in tissues and promote directional migration and infiltration [30, 31]. The expression of proteoglycans and integrins may have promoted the increased infiltration of NK cells in our in vitro assay, which should be further investigated.

In addition to extracellular factors, intracellular components can also affect motility and infiltration. The enhanced infiltration ability and motion in single‐cell tracking of NK cells might be attributed to their increased nuclear flexibility, as has already been suggested for other leukocytes [28]. Similar tendencies have been demonstrated by nuclear lamin A/C data, known as nuclear intermediate filament proteins crucial for cellular functions, including nuclear shape, stiffness, cell motility, chromatin organisation, and gene regulation [28, 35]. Specifically, NK cells display lower levels of lamin A/C, which can be associated with reduced nuclear stiffness. This may improve movement through dense tissue structures. In contrast to NK cells, T cells exhibit higher nuclear stiffness, which may limit their infiltration into microchannels and the tissue environment. These differences in nuclear properties likely contribute to the superior tissue infiltration capacity of NK cells and may enhance their ability to migrate through the endothelial barrier, as nuclear lamin expression has been previously reported to be a crucial factor for cell motion [15, 28, 35, 36, 37, 38]. However, further investigation is required to understand how nuclear flexibility enhances infiltration, which may play a key role in the success of immune cell‐based therapies. The infiltration capacity of NK cells in vivo could be further improved by overexpressing chemokine receptors on the surface of NK cells. Solid tumours are characterised by compact tissue structures that impede immune cell infiltration [39, 40]. Migration and motility towards the tumour are driven by chemokines that bind to specific receptors on the surface of immune cells, thereby inducing chemotaxis [30, 31, 32]. CAR‐NK cells engineered to additionally express CXCR4 have been shown to enhance direct migration towards CXCL12 in tumour‐infiltrated bone marrow compartments in mice in a haematological setting [41, 42, 43].

In addition to the potential for NK cell infiltration, the in vivo persistence of clinically applied NK cell preparations may be a limiting factor compared to the clonal proliferation capacity reported for T cells. However, NK cell persistence can be enhanced using a fourth‐generation CAR construct comprising a cytokine transgene, such as IL‐15, to counteract the shortened lifespan after administration. This was shown to boost NK cell proliferation and persistence in initial clinical trials [16, 17].

We further investigated the motility and target interaction of non‐virally SB engineered NK cells, with a proof‐of‐concept of CD19‐CAR‐NK cells in relation to NT NK cells, as this has not been previously described. The use of SB transposition for CAR‐NK cell generation is a promising approach for non‐viral and efficient CD19‐CAR‐NK cell manufacturing, and a high anti‐tumour response has been demonstrated against B‐ALL cell lines, patient‐derived samples, and in vivo xenograft models [20, 22]. Given their potent cytotoxic function and stable CAR expression, an important question arises as to whether genetic modifications affect their motility compared to NT NK cells. Our findings from microchannels and hyperplastic lymphoid tissue indicated that CD19‐CAR‐NK cells retain motility comparable to that of NT NK cells. We further validated these assumptions using microchannels in the presence of DLBCL cells and within patient‐derived DLBCL biopsy tissues. These tumour tissue slices preserved the complex and immunosuppressive TME, including the vascular structure, stromal elements, and endogenous immune cells [29]. Our observations showed no significant differences between CAR‐NK and NT NK cells, which are consistent with findings in pro‐inflammatory hyperplastic lymphoid tissues. However, while both NK cell preparations exhibited similar velocities when encountering lymphoma cells, CAR‐NK cells displayed a tendency towards a more directed and targeted movement, but decreased infiltration capacity into lymphoma tissue compared to NT NK cells. This suggests that CAR‐NK cells interact more specifically with tumour cells located at the periphery of the tissue sample, resulting in reduced infiltration into the central region. Conversely, NT NK cells exhibited a more undirected motion pattern.

Finally, our results provide new insights into the motility and infiltration capacity of human primary NK cells compared to those of T cells isolated from identical donors. The different functions of these cells in the immune system are reflected in their infiltration patterns. Despite the different extracellular repertoires of chemokine receptors, intracellular factors such as lamins can be assumed to play a role in immune cell motion. Furthermore, our findings indicate that genetic modifications and the integration of artificial CAR into NK cells did not impair their movement. These findings provide insights into the behaviour of CAR‐NK cells in tissues, not only in the context of B cell malignancies but also for the treatment of solid tumours, where the tissue invasion capacity of immune cells plays an important role.

## Author Contributions

**Alina Moter:** data curation (lead), formal analysis (lead), investigation (lead), methodology (lead), validation (lead), visualization (lead), writing – original draft (lead), writing – review and editing (lead). **Sonja Scharf:** data curation (equal), formal analysis (lead), investigation (lead), validation (lead), visualization (lead), writing – review and editing (equal). **Hendrik Schäfer:** data curation (equal), formal analysis (equal), investigation (equal), validation (equal), visualization (equal), writing – review and editing (equal). **Tobias Bexte:** formal analysis (supporting), investigation (equal), methodology (equal), validation (supporting), visualization (supporting), writing – review and editing (equal). **Philipp Wendel:** methodology (equal), writing – review and editing (equal). **Emmanuel Donnadieu:** conceptualization (lead), funding acquisition (lead), project administration (equal), writing – review and editing (equal). **Martin‐Leo Hansmann:** conceptualization (lead), funding acquisition (lead), project administration (lead), resources (lead), supervision (lead), writing – review and editing (equal). **Sylvia Hartmann:** conceptualization (lead), data curation (lead), formal analysis (lead), funding acquisition (lead), project administration (lead), resources (lead), supervision (lead), validation (lead), visualization (lead), writing – original draft (lead), writing – review and editing (lead). **Evelyn Ullrich:** conceptualization (lead), funding acquisition (lead), project administration (lead), resources (lead), supervision (lead), writing – review and editing (lead).

## Ethics Statement

All studies using human material were approved by the local ethical review board (approval no 329/10 and Nr20‐876aV [Frankfurt] and 24‐12237‐BO [Essen]) and were performed in accordance with the Declaration of Helsinki.

## Conflicts of Interest

E.U. has a sponsored research project with Gilead and BMS and acts as a medical advisor for Phialogics and CRIION. The remaining authors declare no conflicts of interest.

## Supporting information


Data S1.

## Data Availability

Imaging data and methods are available from the corresponding author upon reasonable request.
