# Supplementary material for: Migration Dynamics of Human NK Cell Preparations in Microchannels and Their Invasion Into Patient‐Derived Tissue
Source: J Cell Mol Med. 2025 Mar 30;29(7):e70481. doi: 10.1111/jcmm.70481 (PMC11955413; doi:10.1111/jcmm.70481)
Supplement: Supplementary file 1 — Data S1. [file JCMM-29-e70481-s001.zip › jcmm70481-sup-0001-Moter et al_Supplementary Figures_clean version.docx]

Supplementary Figures for

**Migration dynamics of human NK cell preparations in microchannels and their invasion into patient-derived tissue**

**Alina Moter^1,2^, Sonja Scharf^3^, Hendrik Schäfer^3^, Tobias Bexte^1,4,5^, Philipp Wendel^1,2,6,7^, Emmanuel Donnadieu^8^, Martin-Leo Hansmann^9,10^, Sylvia Hartmann^3*^, Evelyn Ullrich^1,2,5,7*^**

*^1^ Goethe University Frankfurt, Department of Pediatrics, Experimental Immunology and Cell Therapy, Frankfurt (Main), Germany
^2^ Goethe University Frankfurt,* *Frankfurt Cancer Institute (FCI), Frankfurt (Main), Germany*

*^3^ Institute of Pathology, University Hospital Essen, University of Duisburg-Essen, Essen, Germany*

*^4^ Institute for Transfusion Medicine and Immunohematology, German Red Cross Blood Service Baden-Württemberg – Hessen, Germany*

*^5^ Goethe University Frankfurt, University Cancer Center Frankfurt (UCT), University Hospital Frankfurt, Frankfurt (Main), Germany*

*^6^ Institute for Organic Chemistry and Biochemistry, Technical University of Darmstadt, Darmstadt, Germany*

*^7^ German Cancer Consortium (DKTK), Partner Site Frankfurt/Mainz and German Cancer Research Center (DKFZ), Heidelberg, Germany*

*^8^ Universite' Paris Cité, CNRS, INSERM, Equipe Labellisée Ligue Contre le Cancer, Institut Cochin, Paris, France*

*^9^ Frankfurt Institute for Advanced Studies (FIAS), Frankfurt (Main), Germany*

*^10^ Institute of General Pharmacology and Toxicology, Goethe University Frankfurt (Main), Germany*

** these authors contributed equally*

**Supplementary Figure 1:**

CD4


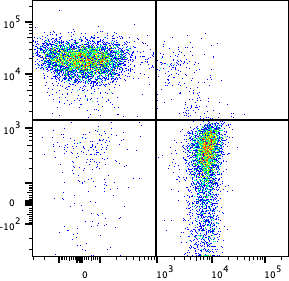


CD8

CD8^+^ T

52.7%

CD4^+^ T

42.8%

CD4^+^CD8^+^ T

2.19%


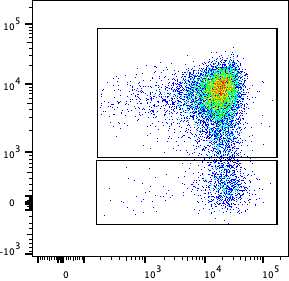


CD16

CD56

CD16^bright^ NK

89.7%

CD16^dim^ NK

9.83%

**A**

**B**

**Supplementary Figure 1: Phenotype of *ex vivo* expanded NK and T cells. (A)** Frequency of CD4^+^ and CD8^+^ CD3^+^ T cells (n=3) and **(B)** of CD16^dim^ and CD16^bright^ CD56^+^ NK cells (n=4) on day 14 post isolation, including exemplary flow cytometry-based dot plots.

**Supplementary Figure 2:**

**
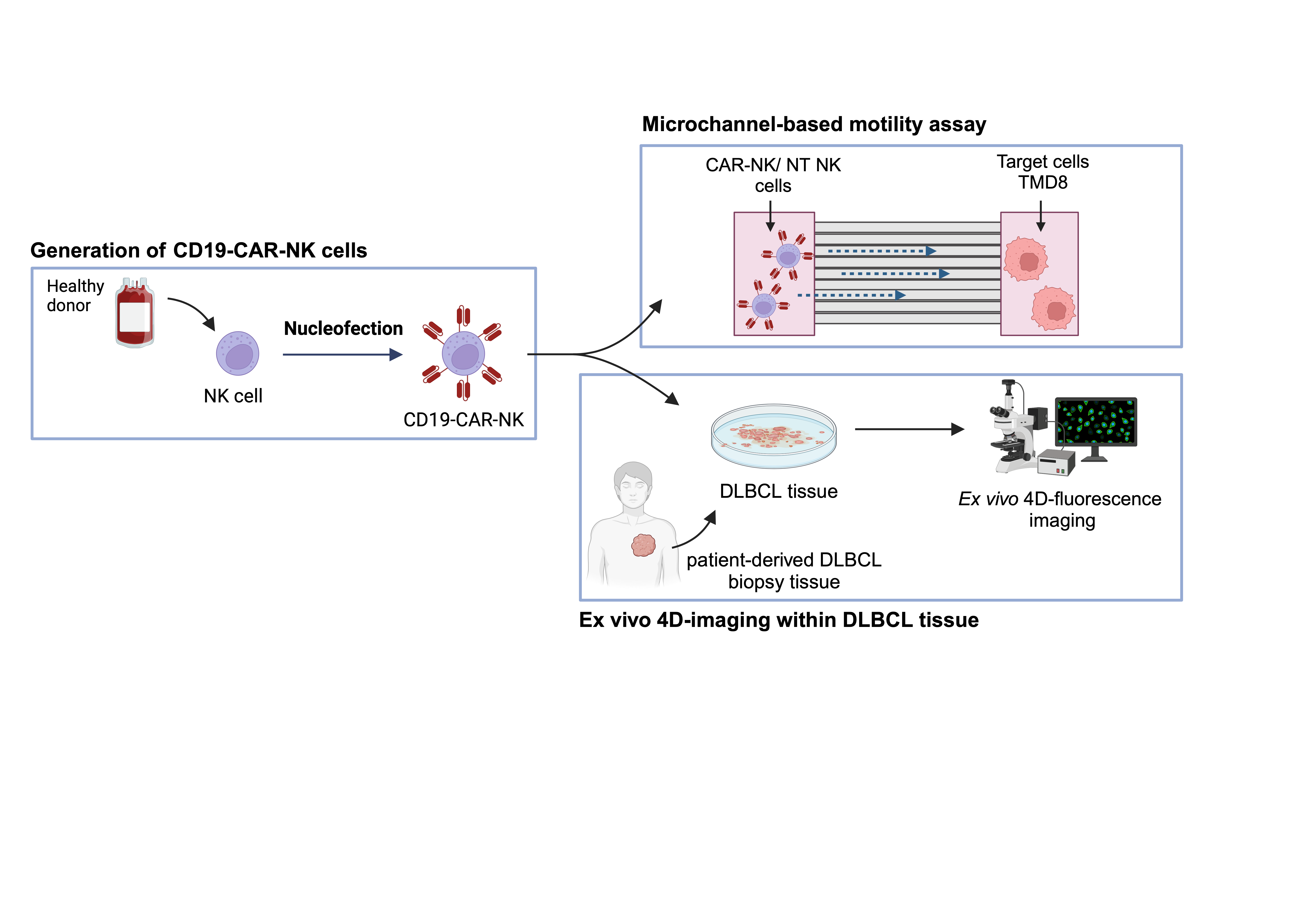
**

**A**

**C**

**B**

**E**

**D**

**F**

**G**

**H**

**Supplementary Figure 2: Motility of CD19-CAR-NK and NT NK cells in presence of TMD8 in microchannel and in patient-derived DLBCL tissue. (A)** Schematic illustration of CD19-CAR-NK cell generation, microchannel based motility assay and *ex vivo* 4D-imaging in DLBCL tissue. **(B)** Number of cells entering the microchannel and **(C)** velocity in the microchannel in presence of TMD8 cells (n=7). Each donor is represented by n=1-4 data points. **(D)** Number of CAR-NK and NT NK cells invading DLBCL tissue (n=2). **(E-H)** Velocity, track length, displacement, and refinement ratio of CAR-NK and NT NK cells in DLBCL tissue (n=2). **(D-H)** One donor is presented by n=5 data points. Microchannel data were statistically analysed using Wilcoxon matched-pairs singed rank test with either all single data points (number of cells entering microchannel) or with the weighted average of each donor (velocity within microchannel). Statistical significance thresholds were set to *p≤ 0.05; ns p> 0.05 (p-values are indicated). For data within DLBCL tissue no statistical analyzation was performed.
