# Supplementary material for: Migration Dynamics of Human NK Cell Preparations in Microchannels and Their Invasion Into Patient‐Derived Tissue
Source: J Cell Mol Med. 2025 Mar 30;29(7):e70481. doi: 10.1111/jcmm.70481 (PMC11955413; doi:10.1111/jcmm.70481)
Supplement: Supplementary file 1 — Data S1. [file JCMM-29-e70481-s001.zip › jcmm70481-sup-0002-Moter et al_Supplementary Information_clean version.docx]

Supplementary information for

**Migration dynamics of human NK cell preparations in microchannels and their invasion into patient-derived tissue**

**Alina Moter^1,2^, Sonja Scharf^3^, Hendrik Schäfer^3^, Tobias Bexte^1,4,5^, Philipp Wendel^1,2,6,7^, Emmanuel Donnadieu^8^, Martin-Leo Hansmann^9,10^, Sylvia Hartmann^3*^, Evelyn Ullrich^1,2,5,7*^**

*^1^ Goethe University Frankfurt, Department of Pediatrics, Experimental Immunology and Cell Therapy, Frankfurt (Main), Germany
^2^ Goethe University Frankfurt,* *Frankfurt Cancer Institute (FCI), Frankfurt (Main), Germany*

*^3^ Institute of Pathology, University Hospital Essen, University of Duisburg-Essen, Essen, Germany*

*^4^ Institute for Transfusion Medicine and Immunohematology, German Red Cross Blood Service Baden-Württemberg – Hessen, Germany*

*^5^ Goethe University Frankfurt, University Cancer Center Frankfurt (UCT), University Hospital Frankfurt, Frankfurt (Main), Germany*

*^6^ Institute for Organic Chemistry and Biochemistry, Technical University of Darmstadt, Darmstadt, Germany*

*^7^ German Cancer Consortium (DKTK), Partner Site Frankfurt/Mainz and German Cancer Research Center (DKFZ), Heidelberg, Germany*

*^8^ Universite' Paris Cité, CNRS, INSERM, Equipe Labellisée Ligue Contre le Cancer, Institut Cochin, Paris, France*

*^9^ Frankfurt Institute for Advanced Studies (FIAS), Frankfurt (Main), Germany*

*^10^ Institute of General Pharmacology and Toxicology, Goethe University Frankfurt (Main), Germany*

** these authors contributed equally*

**Supplementary Methods**

**Human cancer cell lines**

NALM-6 (derived from B-ALL; DSMZ, ACC128) and TMD8 (DLBCL cell line; obtained from Prof. Th. Oellerich, University Hospital Frankfurt) tumour cell lines were splitted twice a week and cultured in RPMI 1640 medium supplemented with GlutaMAX™ (Gibco™), 20% FBS, and 1% penicillin/streptavidin (P/S). The identity of TMD8 cell line was confirmed by short tandem repeats (STR) analysis (data not shown).

**Isolation and cultivation of immune cells**

Freshly isolated NK cells were cultivated in NK-MACS (Miltenyi Biotec) supplemented with 1% NK-MACS® Supplements (Miltenyi Biotec), 5% heat inactivated (h.i.) human plasma (DRK-Blutspendedienst), 1% P/S (Thermo Scientific™) and 120 U/mL IL-15 (Miltenyi Biotec). Immediately after isolation, cells were cultured at a concentration of 2x10^6^ cells/mL. The cells were splitted and adjusted to 1x10^6^ cells/mL every 3-4 days.

Isolation of T cells was performed using the EasySep™ Release Human CD3 Positive Selection Kit (StemCell, Vancouver, Canada). Freshly isolated T cells were cultivated at a concentration of 1x10^6^ cells/mL in RPMI 1640 medium supplemented with GlutaMAX™ (Gibco, Carlsbad, CA, USA), 25 mM HEPES, 10% h.i. human plasma (DRK-Blutspendedienst Baden-Württemberg-Hessen, Frankfurt am Main, Germany), and 1% P/S. On the day of isolation, T cells were stimulated with Dynabeads™ Human T-Activator CD3/CD28 (Gibco, Invitrogen, Carlsbad, CA, USA) at a beads-to-T cell ratio of 1:2 and 50 IU/mL ProleukinS® (Novatis, Basel, Switzerland). On day 6-7 after isolation, CD3/CD28-Dynabeads were removed, and the cell concentration was adjusted to 1x10^6^ cells/mL. T cells were splitted evey 3-4 days and the cell concentration was adjusted to 1x10^6^ cells/mL.

**Purity control of NK and T cell preparations**

T cells were stained with anti-CD3 BUV395 (clone Sk7), anti-CD4 BV786 (clone SK3), and anti-CD8 BUV737 (clone SK1). NK cells were stained with anti-CD56 BV421 (clone NCAM16.2) and anti-CD16 PE-CF594 (clone 3G8) (all BD Biosciences). Samples were measured using the BD FACSCelesta flow cytometer (BD Biosciences) and data were analysed using FlowJo 10.10.0 (FlowJo LLC).

**Cytotoxic function of CD19-CAR-NK cells**

The cytotoxic function of NT and CAR-NK cells against CellTrace™ CFSE-stained NALM-6 cells was assessed using flow cytometry at an E:T ratio of 1:1 after 4 h of co-incubation. Following incubation, cells were stained with DAPI (AppliChem, Darmstadt, Germany), and the specific lysis of tumour cells (CFSE^+^DAPI^+^ cells) was measured using the BD FACSCelesta (BD Biosciences). The data were analysed using FlowJo 10.10.0 (FlowJo LLC).

**Staining and imaging of tissue for ex vivo 4D-imaging**

The analysis of native thick section of hyperplastic lymphatic tissue and DLBCL tissue was performed as previously described.^1–3^ Native tissue was stained with an AF488-coupled anti-CD20 antibody (clone L26, Thermo Fisher Scientific) or anti-CD19 antibody (clone HIB 19, BioLegend).The tissue sections were visualized using a Leica SP8 confocal microscope (Leica Microsystems) while maintaining a steady temperature of 37°C and at regulated oxygen concentrations, controlled by a self-written software. For dynamic imaging, tissue slices were secured with a stainless-steel slice anchor (Warner Instruments) and perfused at a rate of 0.8 mL/min with a solution of RPMI 1640 medium without phenol red, oxygenated with 95% O_2_ and 5% CO_2_. Prior to imaging in the hyperplastic lymphatic tissue, NK and T cells (expanded *ex vivo* for 7-21 days) or CAR-NK and NT NK cells were labeled using CellTrace™ FarRed or CellTrace™ Violet Proliferation Kits (Thermo Fisher Scientific). For imaging in DLBCL tissues, CAR-NK and NT NK cells were stained using CellTrace™ CFSE or CellTrace™ FarRed Proliferation Kits (Thermo Fisher Scientific). Subsequently, 2x10^5^ differently labeled T and NK cells or CAR-NK and NT NK cells were added simultaneously to the tissue slices and incubated for 1 h. For four-dimensional migration analysis stacks of 10-12 sections (z-step = 5 µm) were acquired every 20 s for 15 min at depths up to 80 µm. The Imaris software was then used to extract the motility parameters (speed and distance migrated) of individual immune cells.

**Setting used for confocal microscopy**

The settings used for the confocal microscope were as follows: HC PL APO 63x/1.3 GLYC CORR, Cs2; lasers: 405 nm DMOD Compact, Red 594 nm. The pixel size was 130 nm in each coordinate direction. The z-stack size was set to 0.13 mm and section with a total thickness of 30 µm was analysed.^4^ The analysis of the lamin A/C content per nucleus was performed using the Fiji software.^5^ The DAPI and lamin channels of each image were processed separately. First, we identified the nuclear areas by segmenting the DAPI pixels. The following steps were performed consecutively: Gaussian blur, manual threshold setting, deletion of small and large objects, and computation of the region of interest. Each connected component in the region of interest was considered to be a nucleus object. Subsequently, the lamin channel was segmented by applying a Gaussian blur and setting a manual threshold. The "Analyse Particles" function was then used to measure fraction of lamin-positive pixels per nucleus object.

**Supplementary References**

1. Hartmann S, Scharf S, Steiner Y, et al. Landscape of 4D Cell Interaction in Hodgkin and Non-Hodgkin Lymphomas. *Cancers (Basel)*. 2021;13(20):5208. doi:10.3390/cancers13205208

2. Donnadieu E, Michel Y, Hansmann ML. Live Imaging of Resident T-Cell Migration in Human Lymphoid Tissue Slices Using Confocal Microscopy. *Humana Press*. 2019;1930:75-82. doi:10.1007/978-1-4939-9036-8_10

3. Donnadieu E, Reisinger KB, Scharf S, et al. Landscape of T Follicular Helper Cell Dynamics in Human Germinal Centers. *J Immunol*. 2020;205(5):1248-1255. doi:10.4049/jimmunol.1901475

4. Bein J, Flinner N, Häupl B, et al. T‐cell‐derived Hodgkin lymphoma has motility characteristics intermediate between Hodgkin and anaplastic large cell lymphoma. *J Cell Mol Med*. 2022;26(12):3495-3505. doi:10.1111/jcmm.17389

5. Schindelin J, Arganda-Carreras I, Frise E, et al. Fiji: an open-source platform for biological-image analysis. *Nat Methods*. 2012;9(7):676-682. doi:10.1038/nmeth.2019
