# Supplementary material for: Migration Dynamics of Human NK Cell Preparations in Microchannels and Their Invasion Into Patient‐Derived Tissue
Source: J Cell Mol Med. 2025 Mar 30;29(7):e70481. doi: 10.1111/jcmm.70481 (PMC11955413; doi:10.1111/jcmm.70481)
Supplement: Supplementary file 1 — Data S1. [file JCMM-29-e70481-s001.zip › jcmm70481-sup-0003-Moter et al_Supplementary Movies.docx]

**Supplementary Movies and Methods**

**Supplementary Movie Legend:**

**Supplementary movie S1:** Live cell movie of NK cells in microchannels in the presence of NALM-6. NK cells are shown in blue and NALM-6 cells in black. Monitoring was done for 20h, with time-lapse images taken every 4 min.

**Supplementary movie S2:** Live cell movie of NK and T cells in human patient-derived hyperplastic lymphatic tissue. NK cells are shown in blue, T cells in green and endogenous lymphatic cells in pink. Blue and Green lines demonstrating the tracks of NK and T cells, respectively. Live cell imaging was aquired for 15 min.

**Supplementary movie S3:** Live cell movie of CAR-NK and NT NK cells in human patient-derived hyperplastic lymphatic tissue. CD19-CAR-NK cells are shown in red, NT NK cells in blue and endogenous lymphatic cells in pink. Live cell imaging was performed for 15 min.

**Supplementary Movies**

**Supplementary movie S1:** NK cells and NALM-6 in microchannels

**Supplementary movie S2:** NK and T cells in hyperplastic lymphatic tissue

**Supplementary movie S3:** CD19-CAR NK and NT NK in hyperplastic lymphatic tissue
